# A comparative analysis of single cell small RNA sequencing data reveals heterogeneous isomiR expression and regulation

**DOI:** 10.1038/s41598-022-06876-3

**Published:** 2022-02-18

**Authors:** Christopher Michael Smith, Gyorgy Hutvagner

**Affiliations:** University of Technology Sydney, Sydney, Australia

**Keywords:** Non-coding RNAs, miRNAs, Small RNAs, Molecular biology, Transcriptomics, Sequencing, Next-generation sequencing, Computational biology and bioinformatics

## Abstract

MicroRNAs (miRNAs) are non-coding small RNAs which play a critical role in the regulation of gene expression in cells. It is known that miRNAs are often expressed as multiple isoforms, called isomiRs, which may have alternative regulatory functions. Despite the recent development of several single cell small RNA sequencing protocols, these methods have not been leveraged to investigate isomiR expression and regulation to better understand their role on a single cell level. Here we integrate sequencing data from three independent studies and find substantial differences in isomiR composition that suggest that cell autonomous mechanisms may drive isomiR processing. We also find evidence of altered regulatory functions of different classes of isomiRs, when compared to their respective wild-type miRNA, which supports a biological role for many of the isomiRs that are expressed.

## Introduction

In recent years, technological developments in sequencing, microfluidics and automation have made it possible to collect genomic and whole transcriptomic data from many thousands of cells within a single experiment. Single cell RNA sequencing (scRNA-seq) has revealed extensive heterogeneity in cellular gene expression that traditional approaches such as bulk sequencing could not resolve^1^. Small RNA sequencing, which captures small non-coding RNAs typically missed by standard RNA sequencing, such as microRNAs (miRNAs), PIWI-interacting RNAs (piRNAs), tRNA-derived fragments (tRFs), rRNA-fragments (rRFs) and small nucleolar RNAs (snoRNAs), has more recently been developed for single cell analysis^2^.

One of the most well-known classes of small RNAs are miRNAs, which play a key role in regulating gene expression through post-transcriptional mechanisms that either suppress or degrade other RNAs^3,4^. MiRNA regulation of other RNAs is sequence-specific, facilitated by complementary base pairing between the miRNA and binding sites on their targets. Perfect matching between the seed region, which is the first 2 to 7 or 8 bases of the miRNA from its 5′ end, is typically necessary and in some cases sufficient for gene regulation^5^. This property enables each miRNA to regulate a unique set of targets, and many miRNAs can influence entire gene networks through the regulation of hundreds or even thousands of genes^6^.

IsomiRs are miRNA isoforms which can differ from the wild-type miRNA by as little as one nucleotide^7^. Typically, isomiRs are described or categorized with respect to their difference to a ‘canonical’ miRNA sequence that is somewhat arbitrarily defined by what is most commonly expressed across tissues^8^. Variations can occur at the 5′ or 3′ ends of the miRNA where bases can be added or subtracted, or anywhere within the sequence, including the seed region^9^. 3′ variations are also often distinguished in terms of whether they match their precursor RNA/originating gene sequence or not, as different sequences can be generated during miRNA biogenesis from proteins such as Drosha and Dicer, or by additional factors which can introduce new bases to the 3′ end. A number of studies have shown that many different types of isomiRs can be expressed concurrently and are often more highly expressed than their canonical counterparts^10,11^. There is evidence that different lengths and changes to bases at the 3′ end can alter a miRNAs regulatory activity or stability, whereas shortening or extending the 5′ end of a miRNA is presumed to affect target recognition due to a shift in the seed sequence^7^. However, few studies have investigated the impact that isomiRs have on gene regulation in humans and their relevance in broader contexts such as tissue function and disease remain poorly understood^10,12^.

To our knowledge no study has used single cell small RNA sequencing to investigate isomiR expression or regulation on a single cell level^13^. In this study we analyzed and compared data from three different single cell small RNA sequencing experiments and methodologies and found significant differences in isomiR composition in each study. We observed that isomiR expression was generally similar across individual cells from the same cell culture, however this was not reflected in the Hepatocellular Carcinoma cells sourced from a clinical sample, suggesting that isomiR expression may be highly heterogeneous within the tumor microenvironment and the function of isomiRs may not be fully captured in cell line models. We also attempted to predict isomiR functionality by examining changes in miRNA-targeted transcriptomes, which indicated some isomiRs show different affinities towards their canonical targets and may have distinct functions in single cells.

## Results

### miRNA and IsomiR abundance is highly variable across cell types in the three single cell small RNA-seq protocols

**Figure 1:**
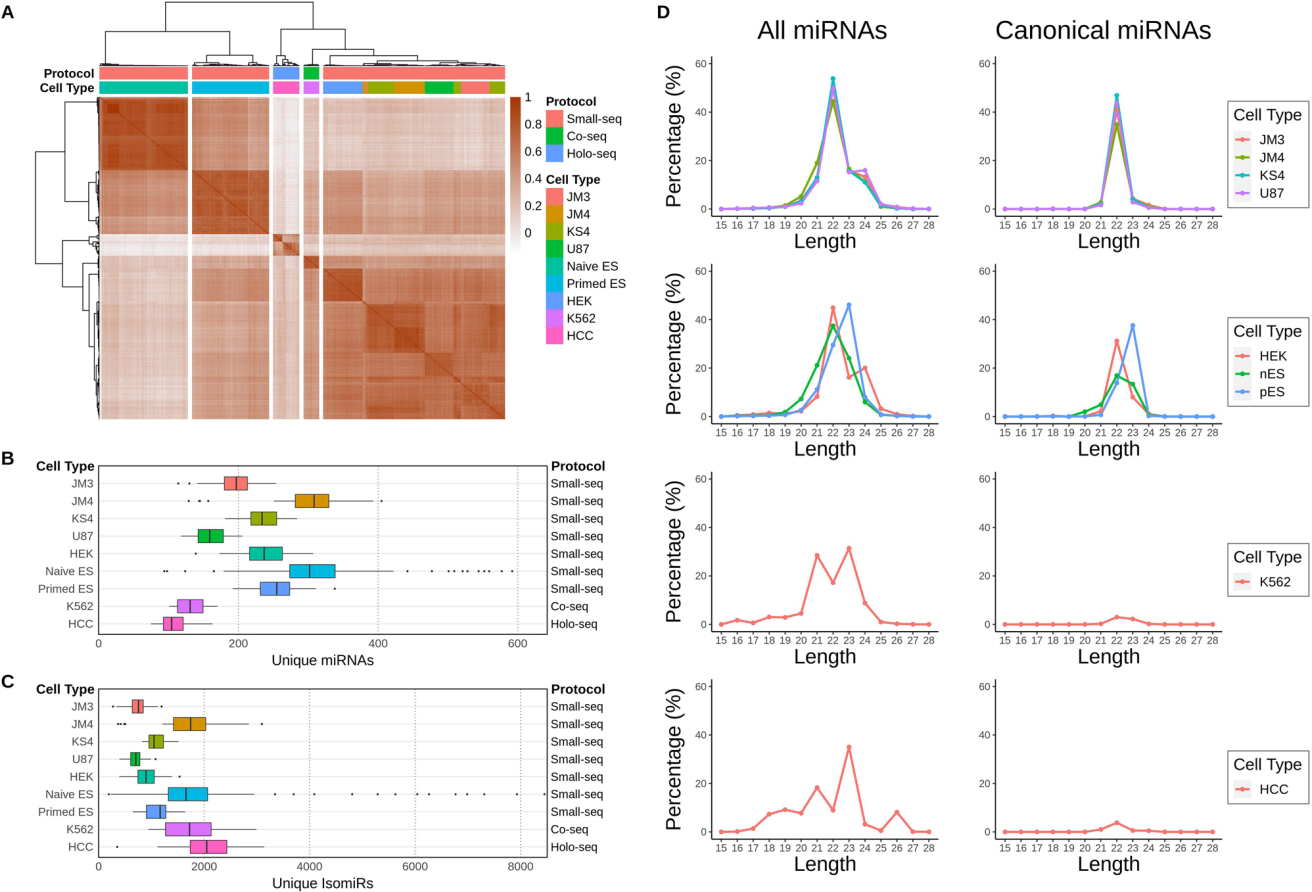
An overview of the small RNA sequencing samples that were analyzed. (**A**) Heatmap showing hierarchical clustering using Spearman correlation of miRNA expression between all samples. (**B**) Number of unique miRNAs detected in each cell type. (**C**) Number of unique isomiRs detected in each cell type. (**D**) Analysis of total miRNA and isomiR length distribution profiles. Distribution of miRNA lengths considering all miRNAs (left) and canonical miRNAs only (right; according to miRbase). For canonical miRNAs, percentages are relative to total miRNA reads. Includes glioblastoma cell lines JM3, JM4, KS4, U87 (Small-seq protocol), embryonic kidney cells (HEK293FT), naïve embryonic (nES), primed embryonic stem cells (pES; Small-seq protocol), K562 cells (Co-seq protocol) and hepatocellular carcinoma cells (HCC; Holo-seq protocol).

To assess miRNA and isomiR expression in different single cell sequencing protocols we analyzed 9 cell types from 3 different studies (Fig. 1A–C and Table 1). This included the seven cell types sequenced in the Small-seq study^2^, which after quality control and filtering included 139 cells from three glioblastoma primary cell cultures, 35 cells from the U87 glioblastoma cell line, 48 cells from the human embryonic kidney cell line HEK293FT, and 107 naïve and 95 primed human embryonic stem cells (Table 1). Additionally, we included the 19 K562 leukemia cells from Wang et al.’s miRNA/mRNA Co-sequencing study (Co-seq)^14^ and 32 hepatocellular carcinoma (HCC) cells isolated from a resected tumor sample and sequenced in the Holo-seq study^15^. Using miRNA expression, we calculated the Spearman correlation between all pairs of cells and clustered them using hierarchical clustering. We found that individual cells from the same cell type typically clustered together, except for several of the glioblastoma cell types which formed mixed clusters likely owing to their similar miRNA profiles. Using the same methodology for read mapping and miRNA annotation for all samples, we found that cells from the Small-seq protocol had a higher number of unique miRNAs. This was despite a larger number of total reads being sequenced from the Co-seq and Holo-seq protocols. However, the cells from the Co-seq and Holo-seq protocols had a higher number of unique isomiRs than most of the other cell types in the Small-seq protocol.

**Table 1:** General features of the single cell small RNA datasets.

Cell type	Cell origin	Culture type/source	Study/protocol	No. of cells included (this study)	Average No. reads mapped to miRNAs (% of Total Reads)	Avg No. UMIs Mapped to miRNAs	Matched RNA-seq data
JM3	Glioblastoma	Primary Cell Culture	Faridani et al.^2^ (Small-seq)	37	6576.46 (0.21%)	2607.30	No
JM4	Glioblastoma	Primary Cell Culture	Faridani et al.^2^ (Small-seq)	42	27,847.19 (0.67%)	7064.76	No
KS4	Glioblastoma	Primary Cell Culture	Faridani et al.^2^ (Small-seq)	60	14,208.52 (0.41%)	4218.68	No
U87	Glioblastoma	Primary Cell Culture	Faridani et al.^2^ (Small-seq)	35	7819.03 (0.18%)	2321.09	No
Naïve Embryonic Stem Cells	Embryonic Stem Cells	Cell Line	Faridani et al.^2^ (Small-seq)	107	43,382.87 (1.03%)	6223.42	No
Primed Embryonic Stem Cells	Embryonic Stem Cells	Cell Line	Faridani et al.^2^ (Small-seq)	95	20,866.96 (0.56%)	3319.39	No
HEK293FT	Embryonic Kidney Cells	Cell Line	Faridani et al.^2^ (Small-seq)	48	8390.38 (0.24%)	2908.21	No
K562	Leukemia	Cell Line	Wang et al.^14^ (Co-seq)	19	25,090.95 (0.38%)	N/A	Yes
Hepatocellular Carcinoma (HCC)	Hepatocellular Carcinoma	Tumor	Xiao et al.^15^ (Holo-seq)	32	31,651.66 (0.38%)	N/A	Yes

Number of cells and average mapping values are according to the mapping and filtering methodology used in this study.

Cell types from each protocol had characteristic miRNA lengths (Fig. 1D) and expression levels (Supplementary Fig. 1). Most of the cell types from the Small-seq study had strong peaks at 22 nt, except for the primed embryonic stem cells which had a high number of 22nt and 23nt miRNAs. A significant proportion of miRNAs, averaged across cell types, in both the Small-seq glioblastoma (42.7–53.7%) and embryonic stem (37.9–52.6%) cell types were annotated as canonical. This was in stark contrast to the Holo-seq and Co-seq protocols where the majority of miRNAs in the HCC and K562 cells were isomiRs, with canonical miRNAs only representing 5.9% and 5.6% of all miRNAs respectively. It is worth noting that miRNA expression for some of the cell types, including the HCC and K562 cells, were dominated by a small number of miRNAs which would have skewed the overall lengths in favor of those miRNA profiles (Supplementary Fig. 1). As the HCC and K562 cell types were sequenced using different protocols from independent labs, it is difficult to know how well the significant differences in isomiR abundance reflect cell specific differences in miRNA processing, maturation, or turnover, and how much is due to technical reasons such as experimental artifacts. Notably, when we compared the single cell data to bulk RNA-seq data from other studies (Supplementary Figs. 2–4), we found the bulk data had length distributions which resembled a more typical 22–23 nt peak in both HCC tumor samples and K562 cells as opposed to the dual peaks observed in the HCC and K562 single cell data (Supplementary Fig. 2).

**Figure 2:**
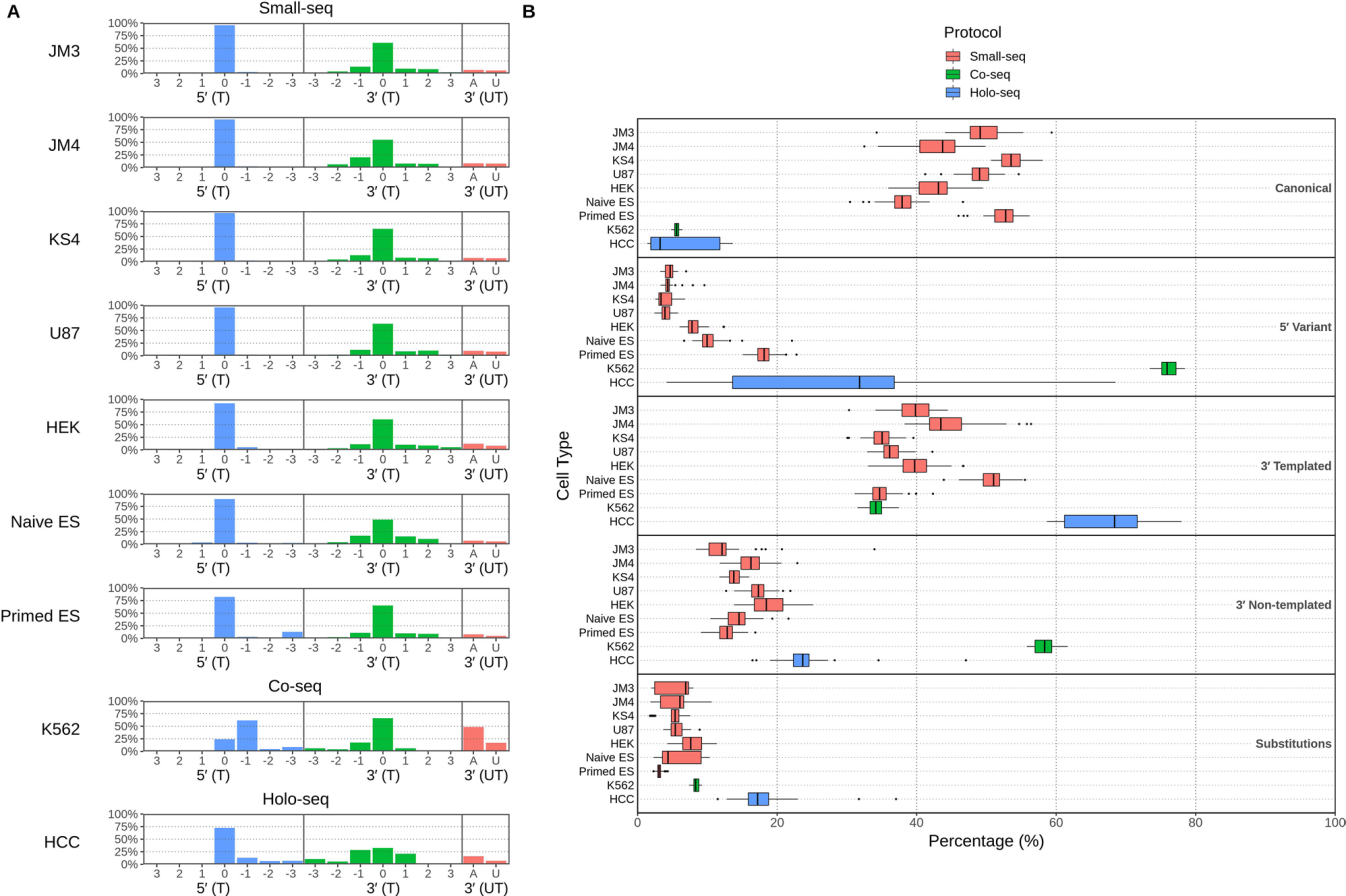
Comparison of relative isomiR expression in each cell type. (**A**) Shows the relative templated proportion of miRNAs according to their 5′ end (blue) or 3′ end (green) templated (T) locations, as well as proportions of miRNAs with non-templated (UT) Adenine (red; A) or Uridine (red; U) additions. (**B**) Box plots constructed from single cell data, showing percentage of total miRNAs belonging to each isomiR category. *ES* Embryonic stem cell.

We then separated isomiRs by their 5′ and 3′ templated locations and calculated the proportion of isomiRs (averaged across single cells in each cell type) starting or ending at each position +/− 3 nucleotides (nts) around the canonical ends (Fig. 2A). Additionally, we included the isomiRs with adenine or uridine bases added to the 3′ end (Fig. 2A) as many studies have shown isomiRs with these additions are more common and can lead to changes in miRNA stability or target recognition^16–18^. 5′ variants which extended the isomiR from the canonical site were almost completely absent in all cells. 5′ variants shorter than the canonical site were also rare, particularly in the Small-seq derived cells, with the exception of primed embryonic stem cells which had a notable amount of isomiRs shortened at the 5′ end by 3 nucleotides (13.0%). However, for the K562 cells, there was a high number of 5′ variants predominantly 1 nt shorter than the canonical site (61.7%), with smaller amounts of 2 and 3 nt shortened isomiRs (4.7% and 8.7% respectively).

With 3′ variants, all cells from the Small-seq and Co-seq protocols showed very similar 3′ templated isomiR profiles according to position, with a strong peak at the canonical site and sharp decline in proportions for the surrounding 1–2 nts. However, HCC cells had nearly equivalent abundances of miRNAs with 3′ canonical sites as well as those shortened or extended by 1 nucleotide. Non-templated 3′ variants containing adenine and/or uridine were present at low levels (12.8–24.2%) in all cell types except K562 cells, where more than half the isomiRs had an untemplated addition (58.2%), most with at least one non-templated adenine (48.8%).

To investigate potential cell-specific mechanisms involved in isomiR expression and processing we labelled isomiRs according to several categories that describe sequence alterations likely to be driven by distinct mechanisms^7^. We then calculated the proportions of every cell’s miRNAs possessing each isomiR alteration (Fig. 2B). Here, we defined the following isomiR categories: canonical (identical sequence to the miRbase annotated miRNA), 5′ variants (altered sequence on the 5′ end, still matching the precursor miRNA sequence), 3′ templated (altered 3′ sequence, but still matching the precursor sequence), 3′ non-templated (altered 3′ sequence with bases not matching its precursor), or substitutions (single nucleotide differences compared to the canonical sequence, excluding 5′ and 3′ ends). Note that each isomiR may contain several sequence alterations and therefore contribute to multiple isomiR categories.

Cells from the Small-seq protocol generally showed similar proportions of isomiR categories, with 3′ templated variants (34.8–50.9%) being the dominant isomiR type (Fig. 2B). Minor differences were observed between cell types which were statistically significant for nearly all categories in this protocol. The majority of miRNAs from the Co-seq K562 cells were 5′ variants (76.1%), with a high proportion of 3′ non-templated variants (58.2%) and similar levels of 3′ templated variants (34.2%) to the Small-seq cells. On the other hand, HCC cells from the Holo-seq protocol produced predominantly 3′ templated (67.3%) and 5′ variants (27.3%), and there was a large increase in variability among single cells across all categories which may reflect the heterogeneity of cells sourced from a tumor sample compared to cell lines or primary cell cultures.

The Small-seq protocol incorporates unique molecular identifiers (UMIs), which tag small RNAs with a unique sequence prior to PCR amplification. UMIs are often used to improve the accuracy of RNA quantification as they can reduce cDNA amplification bias by removing duplicates^19,20^. This enabled us to measure what effect UMI deduplication had on read lengths and the abundance of each type of isomiR (Supplementary Figs. 5–8). A consistent shift in proportions was evident across nearly all isomiR categories and cell types, with a decrease in canonical miRNAs relative to isomiRs after UMI deduplication (Supplementary Fig. 5). Conversely, most isomiR categories had a minor increase in representation, with the largest changes occurring in the naïve and primed embryonic stem cells. For example, in the naïve embryonic stem cells the relative abundance of 5′ altered isomiRs was increased from 10.2 to 17.5% after UMI deduplication, which may have significant implications as 5′ modifications are generally believed to alter a miRNAs’ regulatory targets. Interestingly, UMI deduplication led to a relative decrease in 3′ templated extensions ( +) in the naïve embryonic cells despite increases in all other cell types. Overall, this data indicates that biases introduced during PCR can not only affect miRNA quantification but also the relative abundance of specific isomiRs.

### IsomiR processing is likely regulated by cell autonomous mechanisms

**Figure 3:**
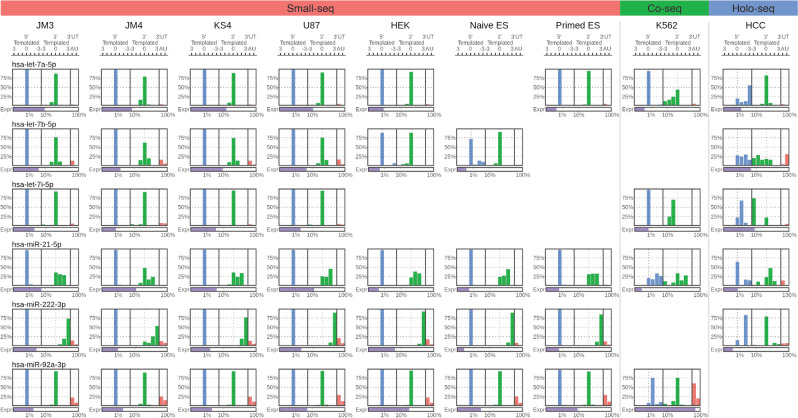
Comparison of 5′ and 3′ isomiRs in miRNAs that are highly expressed across multiple cell types. Shows the relative templated nucleotide position of miRNAs (averaged) at the 5′ end (blue) or 3′ end (green), with respect to the canonical miRNA (according to miRbase). Non-templated additions for Adenine and Uridine are also shown (red). Expression levels (purple) are shown below as percent of total miRNA expression on a log scale. miRNAs with absent plots were either not expressed or did not pass filter criteria. 3′UT: 3′ Non-templated addition. *ES* Embryonic stem cell.

We then re-analyzed isomiRs by their 5′ and 3′ templated locations and adenine/uridine additions, but this time separating isomiRs by their miRNA gene (Fig. 3). We found that different miRNAs exhibited unique patterns of isomiR expression both at the 5′ and 3′ ends.

Expression levels of 3′ templated variants were clearly different between miRNAs from the same cell type (Fig. 3). We did not identify high levels of 5′ variants in any of the Small-seq cells (glioblastoma or embryonic stem cells) when looking at highly expressed miRNAs (Fig. 3). While non-canonical 5′ variants were pervasive in nearly all miRNAs in HCC cells, K562 cells showed a mixture of some miRNAs being heavily modified at the 5′ end and others not modified at all, suggesting this feature is not likely to be a consequence of cell-wide miRNA degradation.

3′ non-templated additions were generally rare in the most highly expressed miRNAs (Fig. 3). One exception was miR-92a-3p, where in K562 cells, 60.8% of miR-92-3p had at least one non-templated adenine and 20.9% with at least one uridine. This coincided with a high overall expression of the miRNA (76.3%) and suggests adenine additions may contribute to the stabilization of miR-92a-3p in K562 cells which would be consistent with previous studies^16^. Adenine and relatively lower levels of uridine additions were observed in all cell types for miR-92a-3p, albeit to a lesser extent than K562 cells.

Overall, we observed that the profiles of the relative abundance of different 5′ and 3′ variants were unique to each miRNA, and it appears that both 5′ and 3′ processing may be driven by mechanisms that distinguish between miRNA species.

Several of the miRNA species were expressed across different cell types, which allowed us to compare how isomiR expression profiles might vary in different biological contexts (Fig. 3 and Supplementary Fig. 9). Again, we observed a high degree of similarity between glioblastoma and embryonic stem cells from the Small-seq protocol, however there were clear examples of miRNAs with very different abundances of their respective isomiRs in the K562 and HCC cells. When comparing let-7a-5p between cell types, all cells except K562 cells predominantly expressed this miRNA matching the 3′ position of the canonical form (73.6–93.7% versus 43.8% in K562 cells), with K562 cells showing a gradual decrease in expression from the canonical position to the shortened 3 nt position that is consistent with degradation of this particular miRNA. This apparent degradation was not evident across many other miRNAs in the K562 cells which suggests this miRNA may have been specifically targeted.

The major differences between cell types in relative isomiR abundance of certain miRNA species suggests cell autonomous mechanisms may be involved in miRNA processing and isomiR expression. However, there was minimal evidence of this when comparing cells from the same cell type (Supplementary Fig. 9), and most of the variation of isomiR abundance within cell types can be explained by differences in miRNA expression, transcriptional stochasticity, and experimental variance.

### IsomiR processing alters regulation of their canonical targets

**Figure 4:**
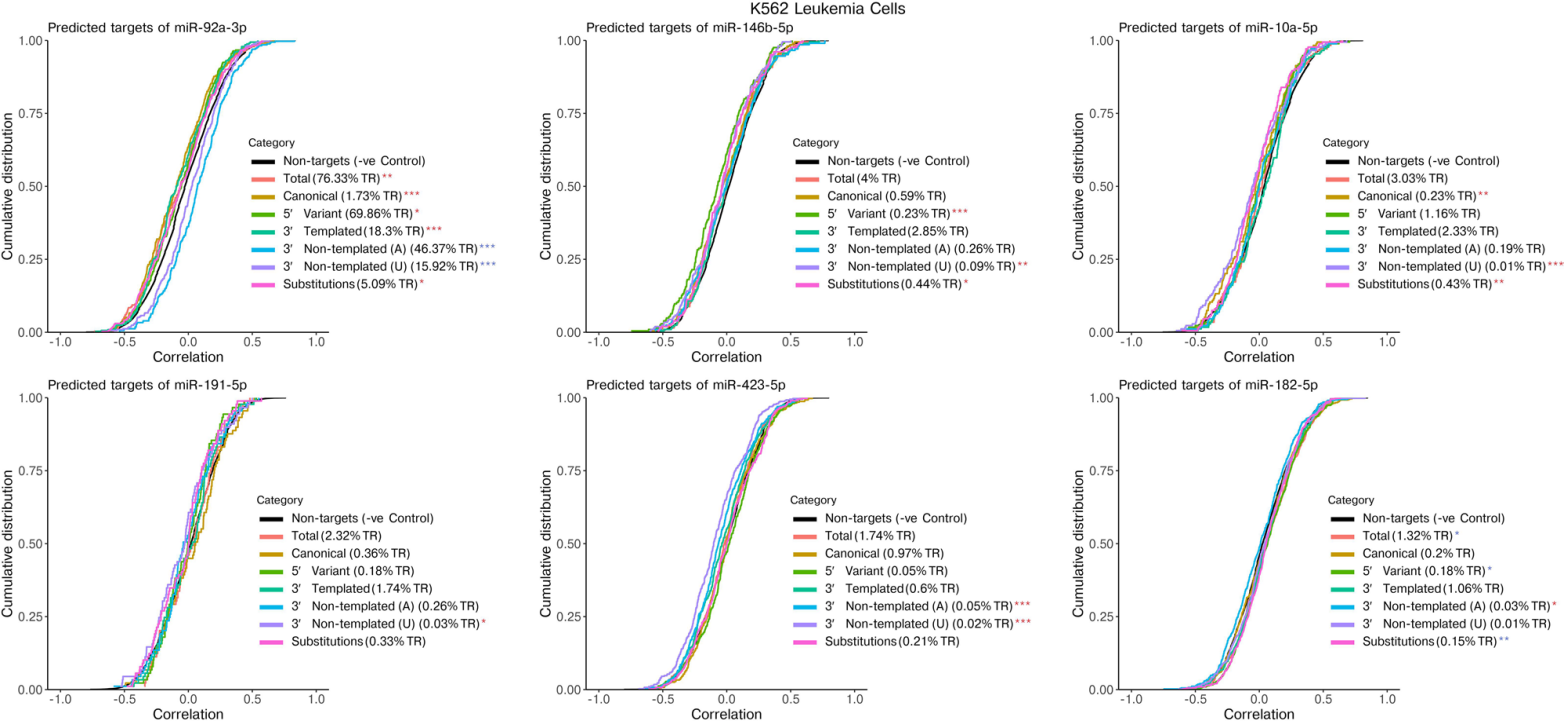
Correlation of expression levels between canonical miRNAs or isomiR categories and the canonical miRNAs predicted targets (mRNAs) in K562 Leukemia cells. In the same miRNA, isomiR categories vary significantly in correlation strength and direction, suggesting these modifications lead to differences in regulatory activity. The 6 highest expressed miRNAs are shown excluding those with low isomiR abundance. For each miRNA, an aggregated list of predicted target mRNAs was collected using miRNAtap and expression of targets was correlated against normalized counts for each isomiR category. Percent of total reads for each isomiR category are shown (averaged across all K562 cells). Non-targets included all remaining mRNAs which were not predicted as a target for that miRNA. Significant differences between each category and Non-targets were calculated using the Kolmogorov–Smirnov test (*p* value < 0.05) and are indicated with a blue (positive correlation) or red (negative correlation) asterisk. TR: Total Reads (mapped to miRNAs).

**Figure 5:**
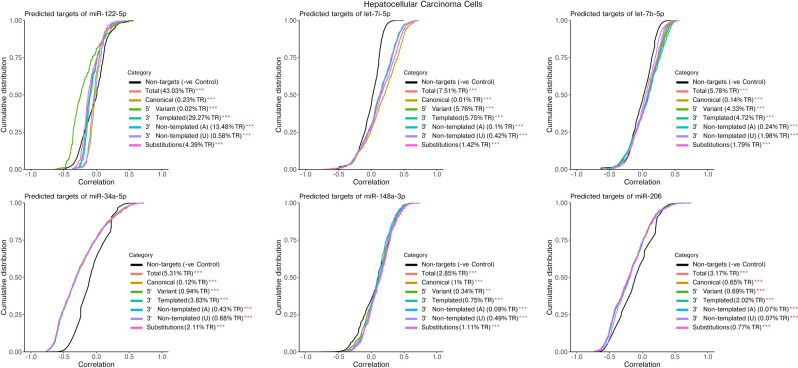
Correlation of expression levels between canonical miRNAs or isomiR categories and the canonical miRNAs predicted targets (mRNAs) in Hepatocellular Carcinoma (HCC) cells. In the same miRNA, isomiR categories vary significantly in correlation strength and direction, suggesting these modifications lead to differences in regulatory activity. The 6 highest expressed miRNAs are shown excluding those with low isomiR abundance. For each miRNA, an aggregated list of predicted target mRNAs was collected using miRNAtap and expression of targets was correlated against normalized counts for each isomiR category. Percent of total reads for each isomiR category are shown (averaged across all HCC cells). Non-targets included all remaining mRNAs which were not predicted as a target for that miRNA. Significant differences between each category and Non-targets were calculated using the Kolmogorov–Smirnov test (*p* value < 0.05) and are indicated with a blue (positive correlation) or red (negative correlation) asterisk. TR: Total Reads (mapped to miRNAs).

The combination of miRNA and mRNA expression data available for the K562 and HCC cells provided us with an opportunity to estimate how different isomiR types could affect regulation of their predicted canonical targets. For this, we generated predicted target lists for the most highly expressed miRNAs using miRNAtap, a tool which aggregates target predictions from multiple sources^21^. Using expression data from each cell, miRNA expression was correlated against expression of each of the miRNAs predicted targets. As a negative control, correlation between each miRNA’s expression and expression of each transcript not predicted to be targeted by the respective miRNA was determined. For each miRNA we also calculated the correlation between expression of isomiRs in each isomiR category and the miRNAs predicted canonical targets, calculated by taking the normalized count of reads mapping to a given miRNA that contained that particular isomiR type. IsomiR categories included—Canonical, 5′ Variant, 3′ Templated, 3′ Non-templated adenine (A), 3′ Non-templated uridine (U) and Substitutions. Results were displayed as a cumulative distribution of all miRNA-gene correlations (Figs. 4, 5). A higher proportion of negatively correlated genes in the target gene set compared to the negative control (non-targets) would cause the line to shift to the left and would suggest that the miRNA was effectively downregulating its targets.

We observed that the relationship between miRNA expression and expression of its predicted canonical targets varied between miRNAs in both cell types (Figs. 4, 5). As was shown in the original Co-seq study, in K562 cells (Fig. 4) there was a stronger negative correlation of the dominantly expressed miR-92a-3p with predicted canonical targets (total) when compared to its correlation with all other transcripts (non-targets)^14^. In contrast, correlation of other highly expressed miRNAs such as miR-146b-5p, miR-10a-5p, miR-191-5p, miR-423-5p and miR-182-5p with their predicted canonical targets (total) were not found to be significantly different to their correlation with all other transcripts (non-targets). In the HCC cells (Fig. 5), several highly expressed miRNAs were more negatively correlated with their predicted targets compared to non-targets, including miR-122-5p, miR-34a-5p and miR-206. Conversely, miR-148a-5p, let-7i-5p, and let-7b-5p were more positively correlated. The results suggest that despite high expression in these cells, many miRNAs may exert a weak or absent regulatory effect on the expression levels of their predicted canonical targets.

When analyzing correlations between expression of canonical miRNAs or each isomiR category with the expression level of the miRNA’s predicted canonical targets it was clear that there were differences in certain isomiR categories for several miRNAs. This was most evident in the K562 cell line where canonical miR-92a-3p and its isomiRs with 5′ Variant or 3′ Templated changes had a negative correlation with its predicted targets. Contrasting this, miR-92a-3p with Non-templated (Adenine) or Non-templated (Uridine) additions were positively correlated (Fig. 4). Interestingly, with miR-146b-5p, 5′ variants had the strongest negative correlation with predicted canonical targets compared to the other isomiR categories, including canonical miRNAs which was not significantly different to non-targets. We did not observe any consistency in the shift in correlations of particular isomiR categories when comparing different miRNAs, indicating that the effect of isomiR types on gene regulation is miRNA specific.

## Discussion

Our analysis of miRNA and isomiR expression in single cell small RNA sequencing data highlighted major differences in the way isomiRs are expressed in different cells. When comparing cell types, one must be careful about concluding that differences in the representation of isomiR types is due to alterations in miRNA processing machinery. It is clear that even within the same cell, each miRNA is uniquely processed, meaning that changes in miRNA expression alone can lead to an overrepresentation of certain isomiR types. This is most evident in the K562 cells where miR-92a-3p dominates miRNA expression. Therefore, it is important that any analysis of isomiR processing not be generalized without considering individual miRNAs. Even within the same cell types, we found variations in the types of isomiRs present in single cells. This was more tightly regulated in cell lines and primary cultures. The high degree of heterogeneity observed in the resected HCC tumor cells suggests that in their native environment, isomiR expression may be far more diverse and potentially also driven by cell-extrinsic factors present in the surrounding microenvironment.

Besides the three studies which each generated and published sequencing data from their own methods, we are not aware of any other studies which have used these methods. Since both the Co-seq and Holo-seq studies only sequenced one human cell type each, and there were no shared cell types across studies, we were unable to ascertain how much of these differences were due to biological or experimental factors. However, we did observe similar trends between the human HCC and mouse embryonic cells from the Holo-seq study, including a low expression of canonical miRNAs and high expression of 5’ isomiRs which clearly distinguished these cells from all of the cell types sequenced in the Small-seq study (Supplementary Fig. 10). Although different protocols were used in each study, many of the principles of small RNA capture and library preparation were identical. All protocols utilized a 3′ ligation step involving a pre-adenylated DNA oligonucleotide, followed by 5′ ligation of an RNA oligonucleotide for small RNA capture. The RNA/DNA-joined molecule was then reverse transcribed and amplified. Therefore, we do not think that the library preparation and sequencing methods can explain the substantial differences in isomiR types across studies. However, we cannot rule out different sample preparation procedures as a potential factor.

There are several challenges that we had to consider when analyzing isomiRs in single cell sequencing data. This included the low number of reads which mapped to miRNAs for each cell, as a high proportion of reads tend to map to ribosomal RNA or do not even map to the human genome. Additionally, the presence of sequencing artefacts introduced during library amplification, as well as biases in ligation efficiencies, can introduce spurious isomiRs or favor isomiRs with certain bases on their 5′ or 3′ ends^22–24^. Low read counts are a typical problem in all single cell sequencing studies, however miRNAs in small RNA-seq often represent an even smaller proportion of total reads than messenger RNAs in RNA-seq. This issue can be further compounded with isomiR studies which separate a miRNA’s reads into individual isomiRs, creating a large number of isomiRs with extremely low counts. This was a particular concern with our analysis of isomiR types and their function of targeting predicted canonical targets, however evidence of altered target regulation by different isomiRs was still present when considering canonical miRNAs and isomiRs with high expression. Therefore, we believe this sufficiently supports the cell autonomous production and function of isomiRs.

Even if present at low frequencies, sequencing artefacts are a particular concern in isomiR studies, as single base alterations can completely redefine an isomiR’s type and interpreted function. Implementing UMI’s into the sequencing library can assist with more accurate measurements of expression levels and enable more confidence in isomiR classification^20,25^. Additionally, ligation biases can be reduced by incorporating random bases at the ends of the adapters which are ligated to RNAs, or on splints which can be introduced to facilitate ligation between adapters and small RNAs^26^.

Previous studies have already investigated the potential for isomiRs to classify cancers with a high degree of success^27,28^. However, these studies only considered cell-normalized expression or the presence/absence of isomiRs. Additional information may be encoded in the relative abundance of isomiRs, that is isomiRs normalized to the number of reads in a cell mapping to their parent miRNA gene. We propose that this approach retains key information about how the cells may be processing each miRNA that may be diluted when normalizing against all other reads or miRNAs. Future studies may be able to leverage this extra layer of information to improve the accuracy of cancer classification.

## Methods

### Data collection

Raw single cell small RNA sequencing data was obtained from the Gene Expression Omnibus (GEO) database under accessions ids GSE81287^2^ (glioblastoma and embryonic cells), GSE114071^14^ (leukemia cells) and the Genome Sequence Archive (Beijing Institute of Genomics) under accession ID CRA001133^15^ (hepatocellular carcinoma cells). Bulk small RNA sequencing data was obtained from the GEO database under accession ids GSE141687^29^ (leukemia cells), GSE76903^30^ and GSE166348^31^ (hepatocellular carcinoma).

### Sequencing data processing

For samples derived from the Small-seq protocol, UMI sequences were removed prior to any adapter removal and appended to the read headers. Adapters were then removed using cutadapt (v2.7) with a minimum overlap of 1 nt and maximum error rate of 0.1 between reads and adapter sequences. After UMI and adapter removal, reads shorter than 15 nucleotides were excluded.

In order to identify duplicate reads for the UMI deduplication comparison, reads were aligned to the human genome (hg38) using bowtie (v1.2.3) with the following parameters: -n 2 -e 120 -l 20 --best. Human-aligned reads were subsequently deduplicated with umitools (v1.0.0) with default settings. Unless specified, all comparisons of isomiR expression between cells were done without deduplication.

### miRNA mapping and annotation

Processed reads were aligned to miRbase (v22) annotated precursor miRNAs using miraligner (v3.4), with the following parameters: -sub 1 -trim 3 -add 3. Reads which successfully aligned to a miRNA were also annotated with any variations to the miRbase defined mature sequence.

The following isomiR categories were defined: Canonical—miRNAs with a perfect match to the miRbase mature sequence, 5′ Variant—miRNAs differing at the 5′ end with respect to the miRbase mature sequence, 3′ Templated—miRNAs deviating in length to the miRbase mature sequence but still matching the precursor miRNA sequence, 3′ Non-templated—miRNAs which did not match the precursor miRNA sequence at the 3′ end, and Substitution—miRNAs containing a maximum of 1 mismatch to the miRbase mature sequence, excluding variations at the 5′ or 3′ ends. Categories were then assigned any alignments which contained their respective isomiR type and were used for measuring their proportions relative to the total number of annotated miRNAs. Cells containing less than 1000 reads mapping to miRNAs (prior to any UMI deduplication) were excluded from further analysis. Figures were generated with the R packages ggplot2 and pheatmap^32^.

### Correlation of miRNA and IsomiR expression with predicted targets

For building miRNA predicted target lists, the miRNAtap (v1.20) package was used to aggregate targets across the five supported algorithms (DIANA, Miranda, PicTar, TargetScan and miRDB) using the ‘minimum’ method and only considering targets predicted by 2 or more algorithms. The Pearson correlation between miRNA and gene expression across all cells was calculated and used for plotting each miRNA with its targets.

## Supplementary Information

Supplementary Information.

## References

[1] Hwang B, Lee JH, Bang D (2018). Single-cell RNA sequencing technologies and bioinformatics pipelines. Exp. Mol. Med..

[2] Faridani OR (2016). Single-cell sequencing of the small-RNA transcriptome. Nat. Biotechnol..

[3] Bartel DP (2004). MicroRNAs. Cell.

[4] O’Brien J, Hayder H, Zayed Y, Peng C (2018). Overview of MicroRNA biogenesis, mechanisms of actions, and circulation. Front. Endocrinol. (Lausanne)..

[5] Brennecke J, Stark A, Russell RB, Cohen SM (2005). Principles of microRNA-target recognition. PLoS Biol..

[6] Bartel DP (2009). MicroRNAs: Target recognition and regulatory functions. Cell.

[7] Neilsen CT, Goodall GJ, Bracken CP (2012). IsomiRs—The overlooked repertoire in the dynamic microRNAome. Trends Genet..

[8] Budak H, Bulut R, Kantar M, Alptekin B (2016). MicroRNA nomenclature and the need for a revised naming prescription. Brief. Funct. Genomics.

[9] Guo L, Chen F (2014). A challenge for miRNA: Multiple isomiRs in miRNAomics. Gene.

[10] Haseeb A, Makki MS, Khan NM, Ahmad I, Haqqi TM (2017). Deep sequencing and analyses of miRNAs, isomiRs and miRNA induced silencing complex (miRISC)-associated miRNome in primary human chondrocytes. Sci. Rep..

[11] Salem O (2016). The highly expressed 5’isomiR of hsa-miR-140-3p contributes to the tumor-suppressive effects of miR-140 by reducing breast cancer proliferation and migration. BMC Genom..

[12] Yu F (2017). Naturally existing isoforms of miR-222 have distinct functions. Nucleic Acids Res..

[13] Mukherji S (2011). MicroRNAs can generate thresholds in target gene expression. Nat. Genet..

[14] Wang N (2019). Single-cell microRNA-mRNA co-sequencing reveals non-genetic heterogeneity and mechanisms of microRNA regulation. Nat. Commun..

[15] Xiao Z (2018). Holo-Seq: Single-cell sequencing of holo-transcriptome. Genome Biol..

[16] D’Ambrogio A, Gu W, Udagawa T, Mello CC, Richter JD (2012). Specific miRNA stabilization by Gld2-catalyzed monoadenylation. Cell Rep..

[17] Gutiérrez-Vázquez C (2017). 3′ Uridylation controls mature microRNA turnover during CD4 T-cell activation. RNA.

[18] Yang A (2019). 3′ Uridylation confers miRNAs with non-canonical target repertoires. Mol. Cell.

[19] Islam S (2014). Quantitative single-cell RNA-seq with unique molecular identifiers. Nat. Methods.

[20] Fu Y, Wu PH, Beane T, Zamore PD, Weng Z (2018). Elimination of PCR duplicates in RNA-seq and small RNA-seq using unique molecular identifiers. BMC Genom..

[21] Pajak, M., Simpson, T. I. miRNAtap: miRNAtap: microRNA Targets—Aggregated Predictions. *R package version 1.20.0* (2019).

[22] Sena JA (2018). Unique Molecular Identifiers reveal a novel sequencing artefact with implications for RNA-Seq based gene expression analysis. Sci. Rep..

[23] Wright C (2019). Comprehensive assessment of multiple biases in small RNA sequencing reveals significant differences in the performance of widely used methods. BMC Genom..

[24] Jayaprakash AD, Jabado O, Brown BD, Sachidanandam R (2011). Identification and remediation of biases in the activity of RNA ligases in small-RNA deep sequencing. Nucleic Acids Res..

[25] Mitchell K (2020). Benchmarking of computational error-correction methods for next-generation sequencing data. Genome Biol..

[26] Maguire S, Lohman GJS, Guan S (2020). A low-bias and sensitive small RNA library preparation method using randomized splint ligation. Nucleic Acids Res..

[27] Telonis AG (2016). Knowledge about the presence or absence of miRNA isoforms (isomiRs) can successfully discriminate amongst 32 TCGA cancer types. Nucleic Acids Res..

[28] Lan C, Peng H, McGowan EM, Hutvagner G, Li J (2018). An isomiR expression panel based novel breast cancer classification approach using improved mutual information. BMC Med. Genom..

[29] Kania EE (2020). Hsa-miR-9-3p and hsa-miR-9-5p as post-transcriptional modulators of DNA topoisomerase IIa in human leukemia K562 cells with acquired resistance to etoposide. Mol. Pharmacol..

[30] Yang Y (2017). Recurrently deregulated lncRNAs in hepatocellular carcinoma. Nat. Commun..

[31] Zhao J (2021). Epigenetic silencing of miR-144/451a cluster contributes to HCC progression via paracrine HGF/MIF-mediated TAM remodeling. Mol. Cancer.

[32] Wickham H (2016). ggplot2: Elegant Graphics for Data Analysis.

