# Supplementary material for: A comparative analysis of single cell small RNA sequencing data reveals heterogeneous isomiR expression and regulation
Source: Sci Rep. 2022 Feb 18;12:2834. doi: 10.1038/s41598-022-06876-3 (PMC8857176; doi:10.1038/s41598-022-06876-3)
Supplement: Supplementary file 1 — Supplementary Information. [file 41598_2022_6876_MOESM1_ESM.docx]

## Supplementary Figures


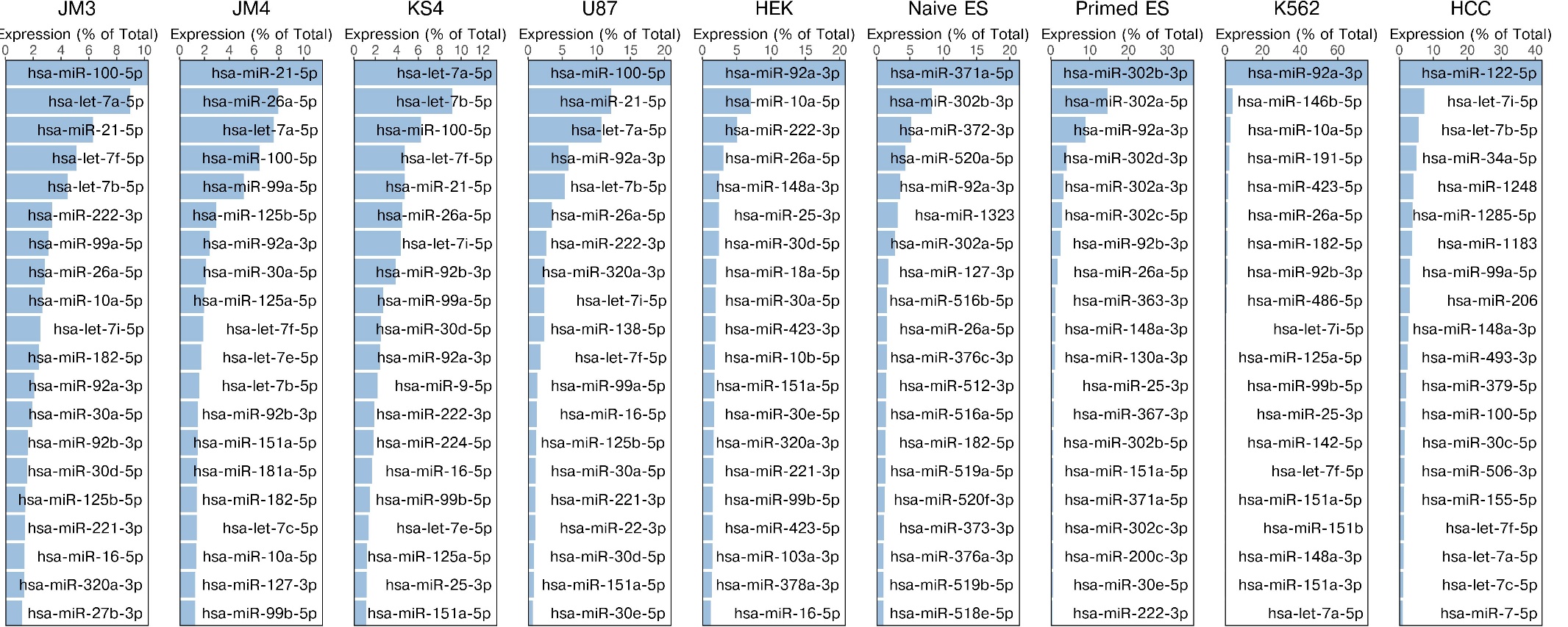


#### **Supplementary Figure 1.** Top 20 expressed miRNAs (combined canonical and isomiR expression) in each cell type from single cell small RNA sequencing studies. Includes the glioblastoma primary cultures/cell lines JM3, JM4, KS4 and U87, human embryonic kidney cell line HEK293 (HEK), naïve and primed embryonic stem (ES) cells, the K562 leukemia cell line and hepatocellular carcinoma cells (HCC).


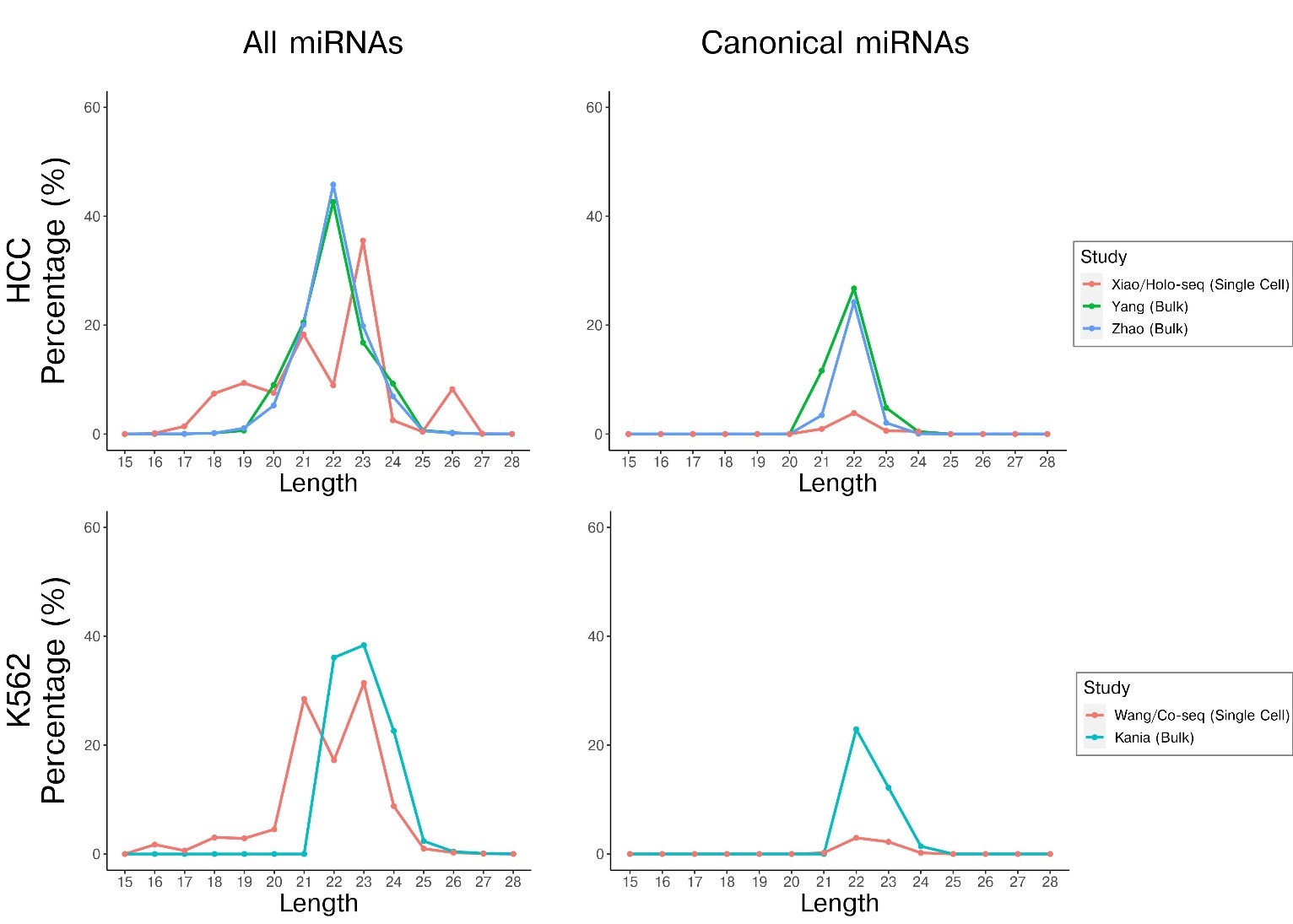


#### **Supplementary Figure 2.** Comparison of total miRNA and isomiR length distribution profiles between single cell and bulk RNA-seq datasets from independent studies. Includes HCC tumor and K562 cells. Distribution of miRNA lengths considering all miRNAs (left) and canonical miRNAs only (right; according to miRbase). For canonical miRNAs, percentages are relative to total miRNA reads.


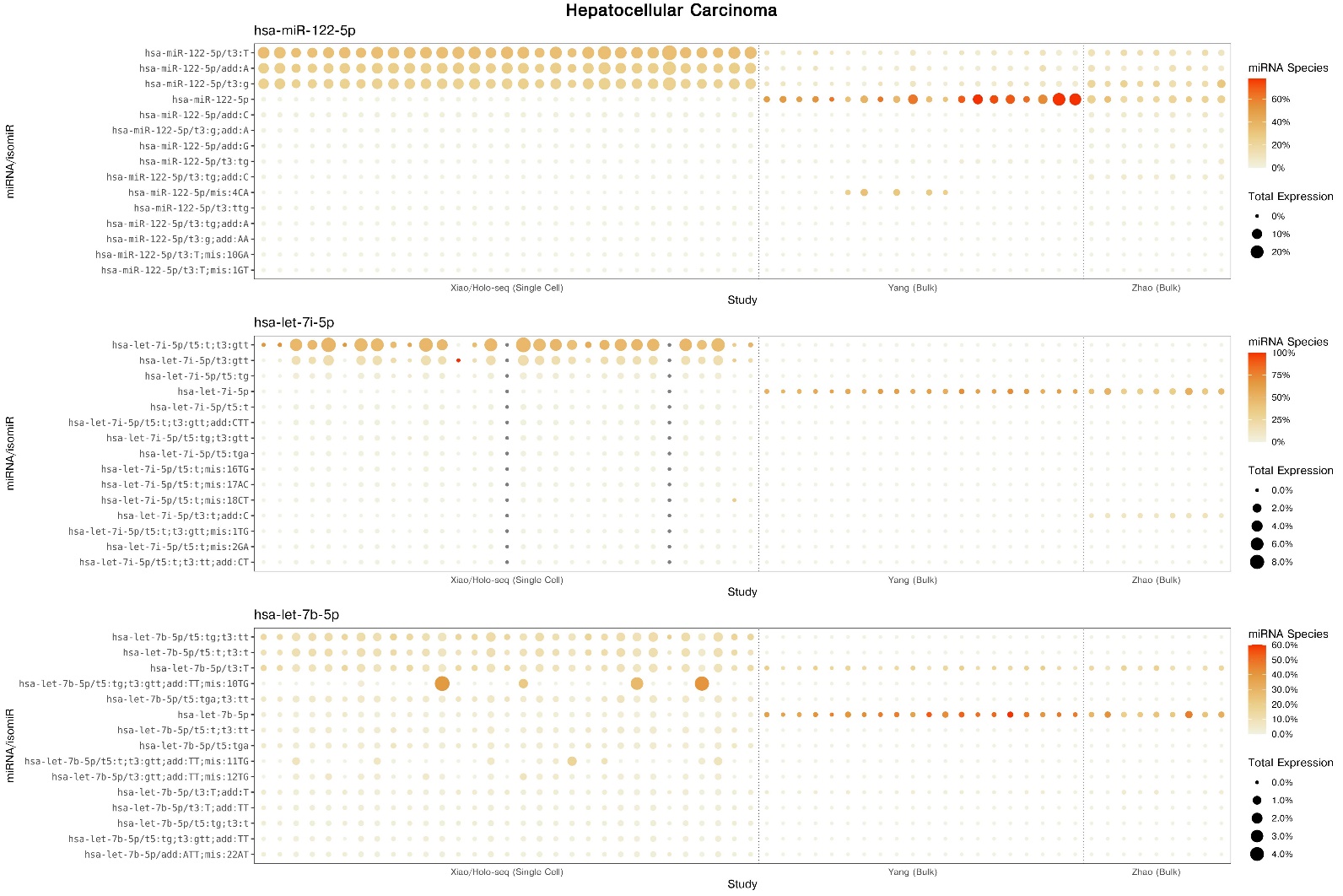


#### **Supplementary Figure 3.** Expression and relative abundance of isomiRs in Hepatocellular Carcinoma single cell and bulk smRNA-seq data from independent studies. Shows each isomiRs expression (rows) across individual cells (columns), with respect to their relative abundance of their miRNA gene (dot color) and the isomiRs expression normalized to total miRNAs for each cell (dot size). Top 15 isomiRs for the 3 highest expressed miRNAs in the single cell dataset are displayed. Cells which did not have any reads mapping to the miRNA are indicated by grey circles.


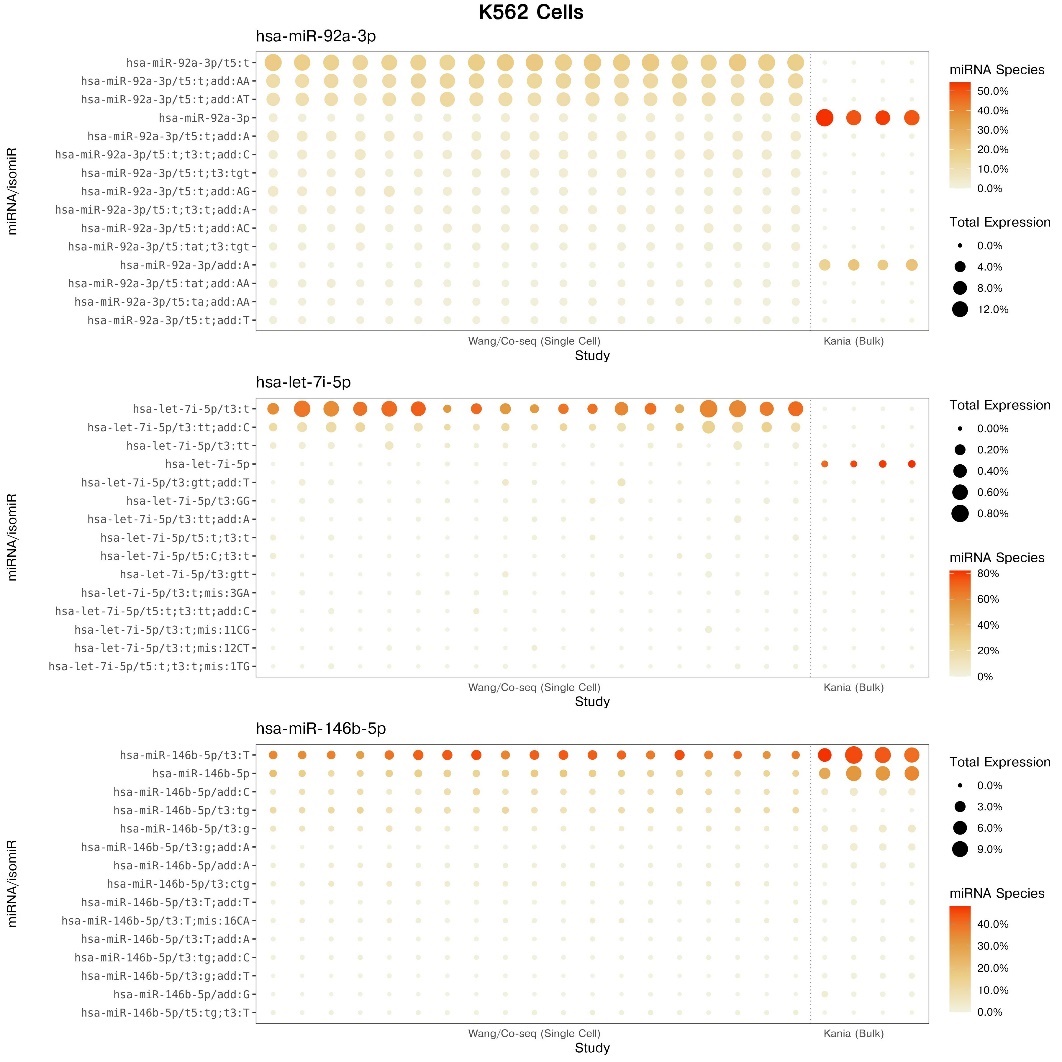


#### **Supplementary Figure 4.** Expression and relative abundance of isomiRs in K562 Leukemia single cell and bulk smRNA-seq data from independent studies. Shows each isomiRs expression (rows) across individual cells (columns), with respect to their relative abundance of their miRNA gene (dot color) and the isomiRs expression normalized to total miRNAs for each cell (dot size). Top 15 isomiRs for 3 highly expressed miRNAs in the single cell dataset are displayed. Cells which did not have any reads mapping to the miRNA are indicated by grey circles.


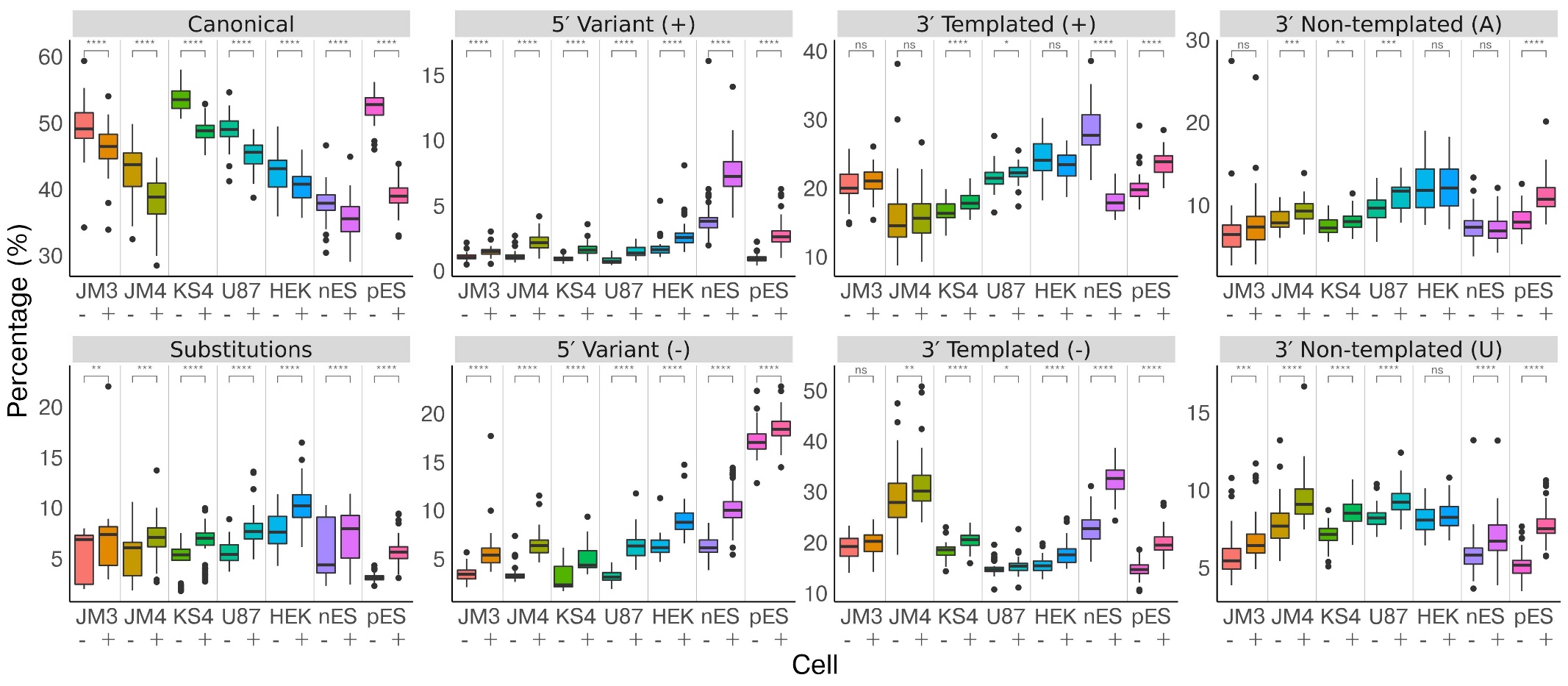


#### **Supplementary Figure 5.** Impact of UMI deduplication on isomiR types. For each cell non-deduplicated reads (-) are shown next to deduplicated reads (+). ns: Not significant. Includes the glioblastoma primary cultures/cell lines JM3, JM4, KS4 and U87, human embryonic kidney cell line HEK293 (HEK), naïve embryonic stem (nES) and primed embryonic stem (pES) cells, the K562 leukemia cell line and hepatocellular carcinoma cells (HCC). 5′ Variant and 3′ Templated isomiRs are also separated by those longer (top, +) or shorter (bottom, -) than the canonical form.


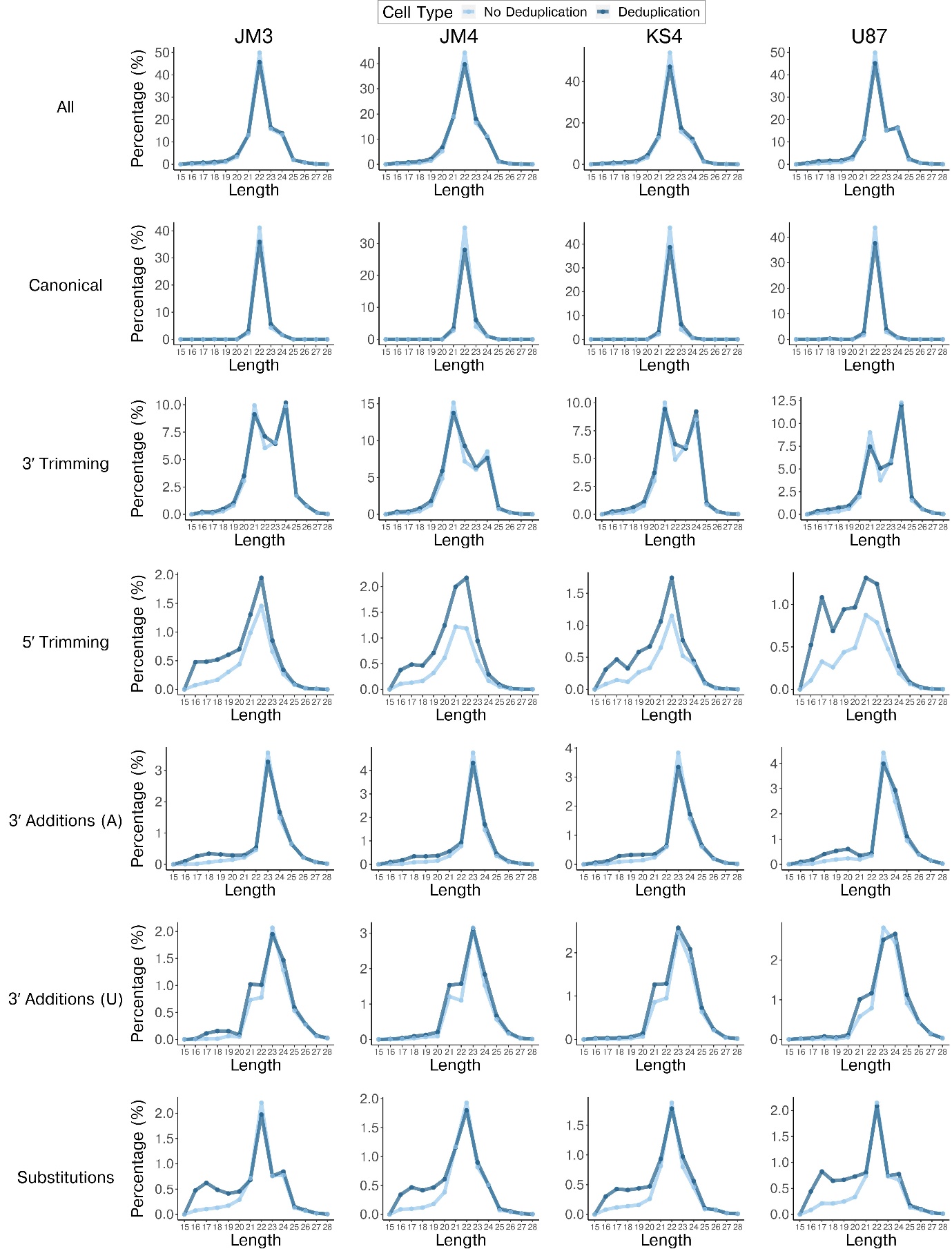


#### **Supplementary Figure 6.** Impact of UMI deduplication on miRNA length distributions in glioblastoma primary cultures and cell lines (JM3, JM4, KS4 and U87). Light blue: Read lengths without deduplication. Dark blue: Read lengths with UMI deduplication.


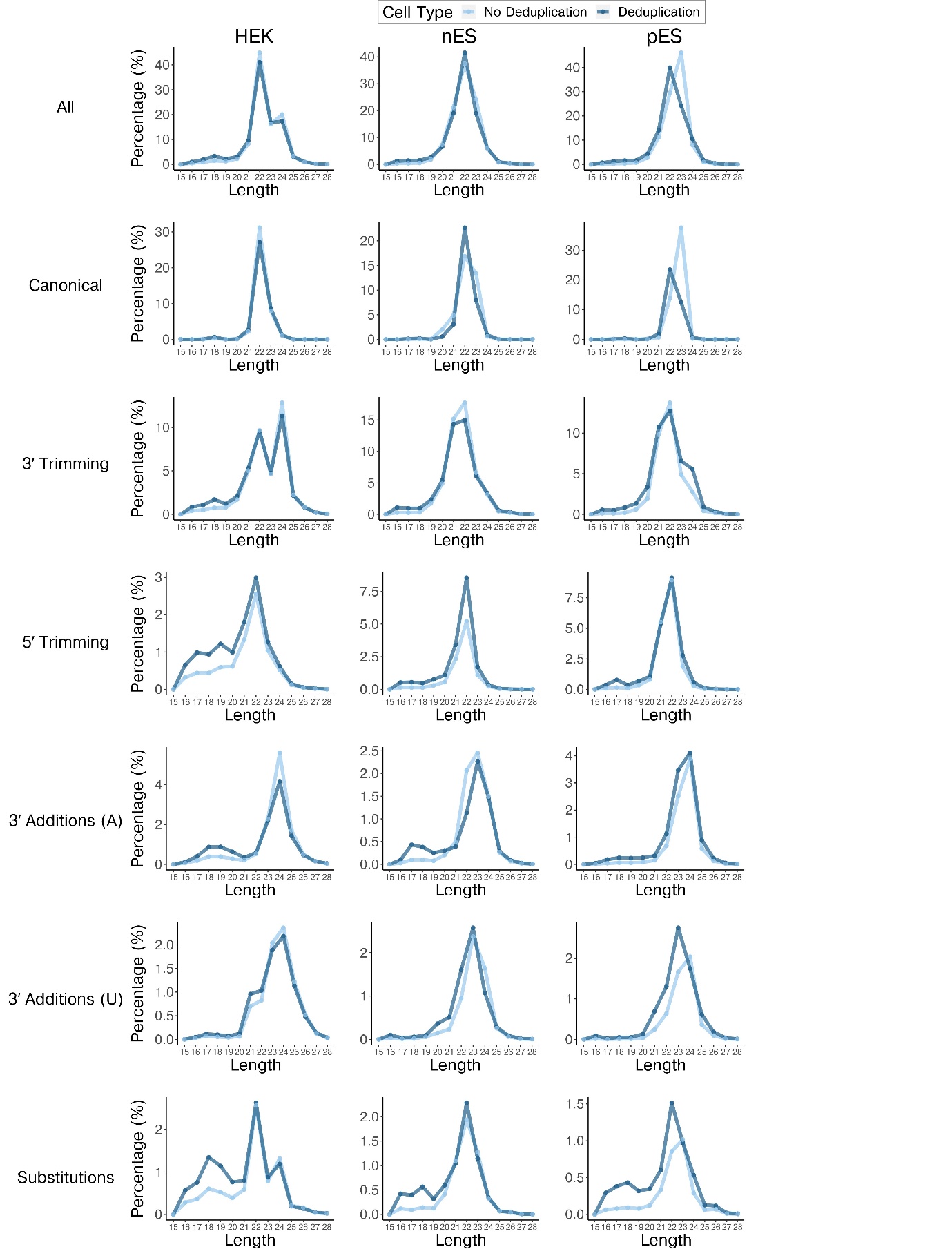


#### **Supplementary Figure 7.** Impact of UMI deduplication on miRNA length distributions in the HEK293 cell line, naïve embryonic stem (nES) and primed embryonic stem (pES) cells. Light blue: Read lengths without deduplication. Dark blue: Read lengths with UMI deduplication.


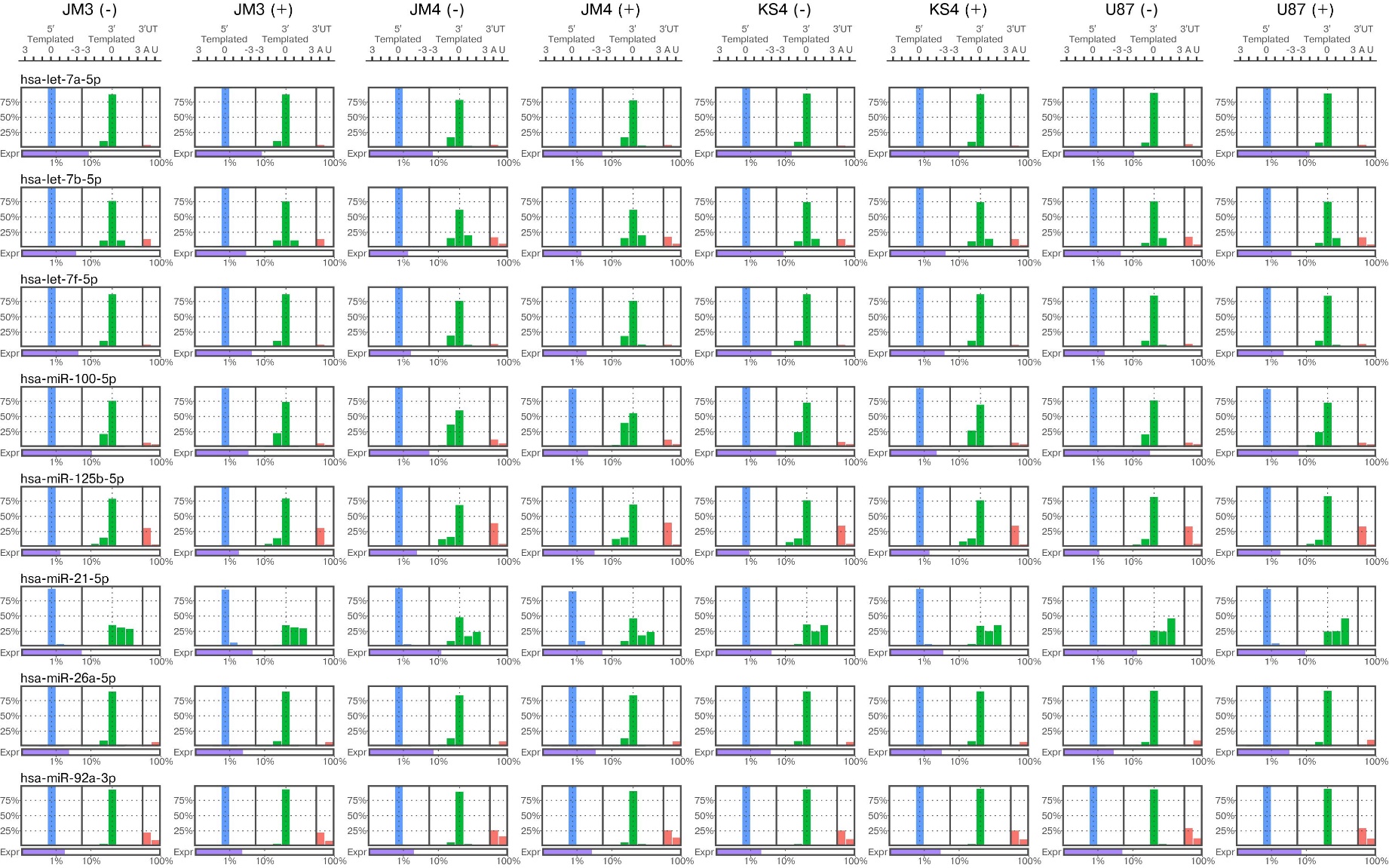


#### **Supplementary Figure 8.** Comparison of 5′ and 3′ isomiR variations between averaged non-deduplicated (-) and deduplicated (+) glioblastoma cells, for selected miRNAs.


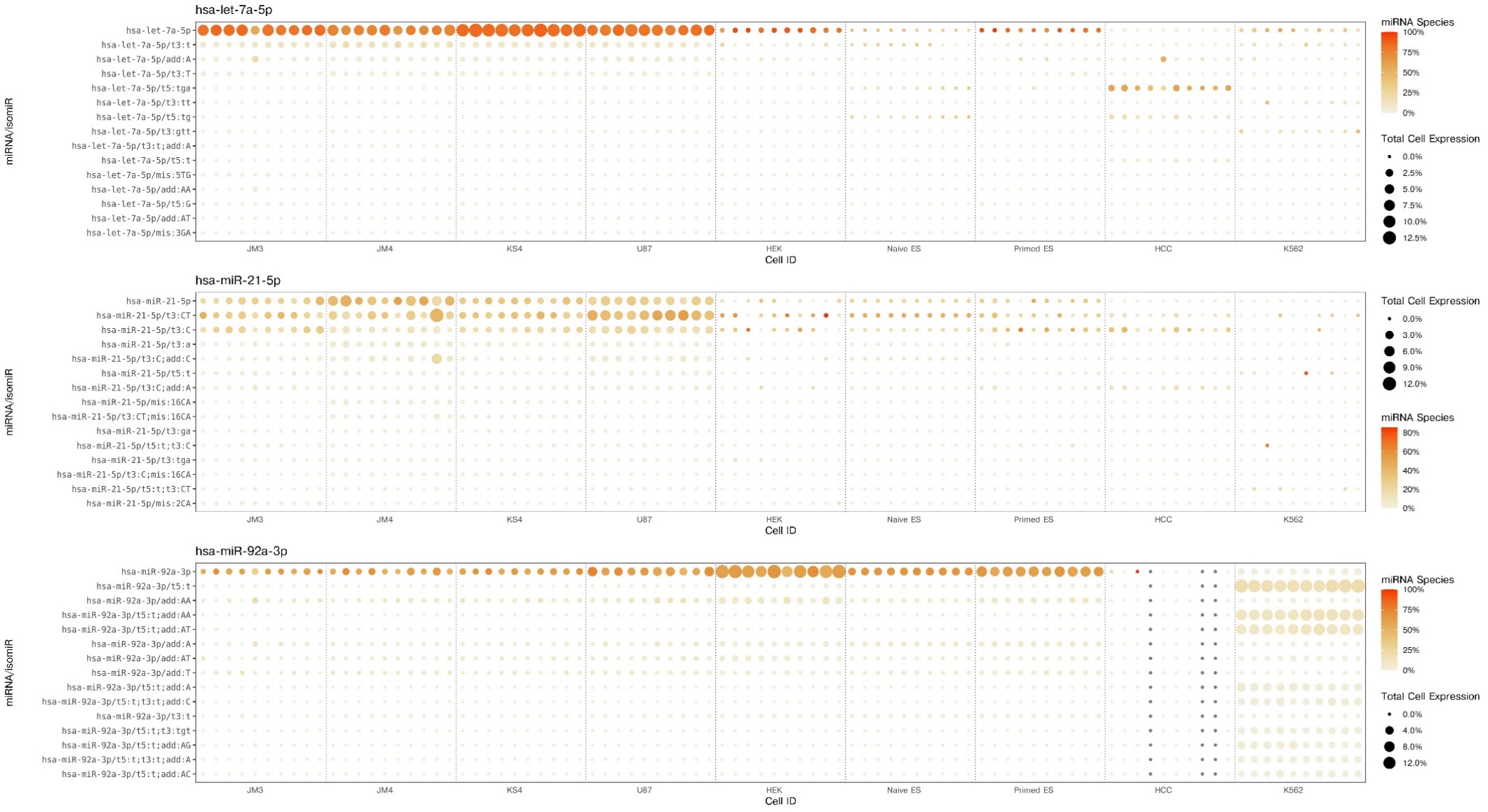


**Supplementary Figure 9.** Expression and relative abundance of isomiRs in single cells. Shows each isomiRs expression (rows) across individual cells (columns), with respect to their relative abundance of their miRNA gene (dot color) and the isomiRs expression normalized to total miRNAs for each cell (dot size). Top 15 expressed isomiRs for let-7a-5p, miR-21-5p and miR-92a-3p are displayed. 10 cells from each cell type are included. Cells which did not have any reads mapping to the miRNA are indicated by grey circles.


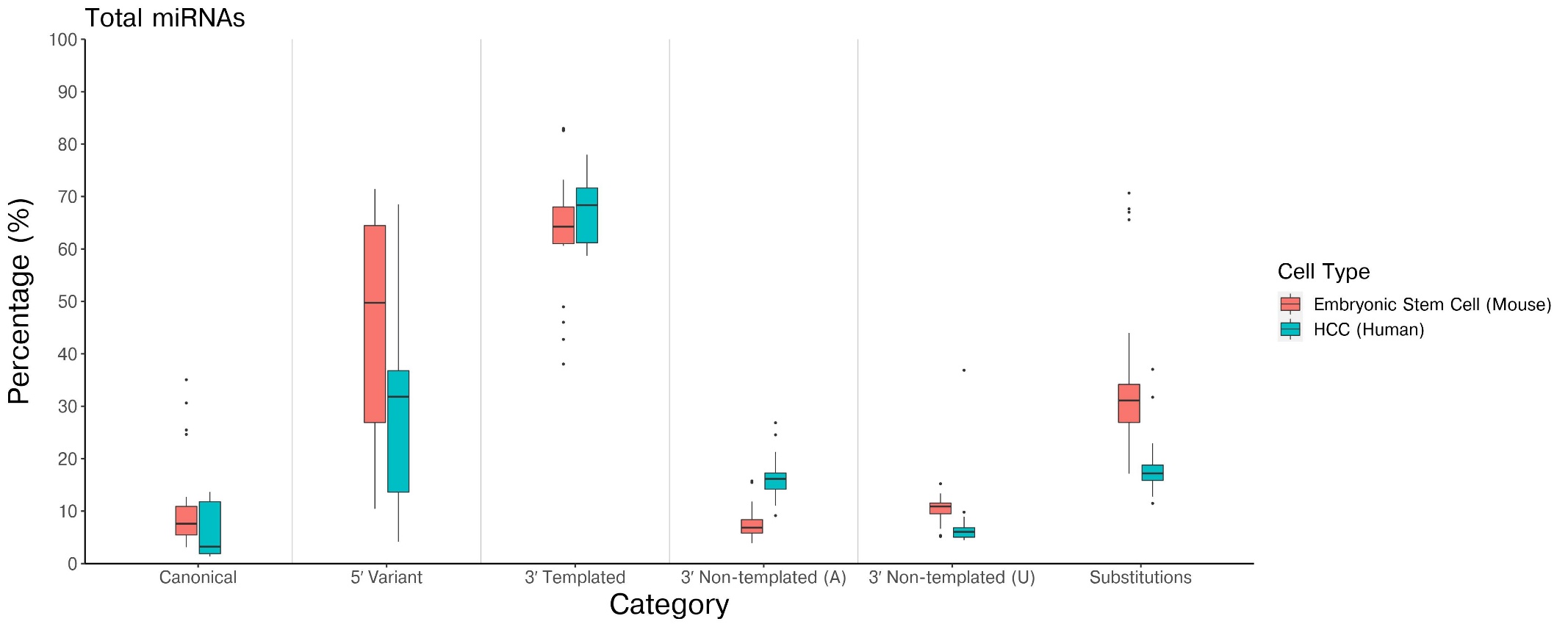


#### **Supplementary Figure 10.** Comparison of relative isomiR expression in mouse embryonic stem cells (red) and human hepatocellular carcinoma cells (blue) from the Holo-seq study. Box plots constructed from single cell data, showing percentage of total miRNAs belonging to each isomiR category.
